# Exploring corrosion protection properties of alkyd@lanthanide bis-phthalocyanine nanocomposite coatings

**DOI:** 10.1039/c7ra09804a

**Published:** 2018-01-09

**Authors:** M. A. Deyab, R. Słota, E. Bloise, G. Mele

**Affiliations:** Egyptian Petroleum Research Institute (EPRI) PO Box 11727, Nasr City Cairo Egypt hamadadeiab@yahoo.com; Faculty of Chemistry, Opole University ul. Oleska 48 45-052 Opole Poland; Department of Engineering for Innovation of University of Salento via Arnesano 73100 Lecce Italy giuseppe.mele@unisalento.it

## Abstract

Organic coatings have been widely used to protect carbon steel pipelines from external corrosion; however, they often suffer from permeability and weak adhesion. Here we show that synthetic lanthanide bis-phthalocyanine complexes, LnPc_2_ (Ln = lanthanide metal, Pc = C_32_H_16_N_8_ denotes the phthalocyanine ligand) can be used to form new nanocomposite coatings to provide corrosion protection to the underlying carbon steel pipelines. Electrochemical studies (EIS and potentiodynamic polarization) showed that the incorporation of LnPc_2_ compound (PrPc_2_, SmPc_2_ and HoPc_2_) additives with alkyd coating, leads to a significant increase in the corrosion resistance of carbon steel in 0.5 M HCl solution. The alkyd@LnPc_2_ nanocomposite coatings absorb very low water volumes, when compared to the neat alkyd coating. LnPc_2_ compounds allowed enhancing the pull-off adhesion of coatings performance from 3.34 MPa to 19.94 MPa. The efficiency of alkyd@HoPc_2_ coating appears higher than that of alkyd@PrPc_2_ and alkyd@SmPc_2_ coatings. The protective properties of alkyd@LnPc_2_ coatings were confirmed by SEM, TGA, scratch hardness, impact resistance, bend test and contact angle analysis.

## Abstract 2

Organic coatings have been widely used to protect carbon steel pipelines from external corrosion; however, they often suffer from permeability and weak adhesion.

## Graphical abstract

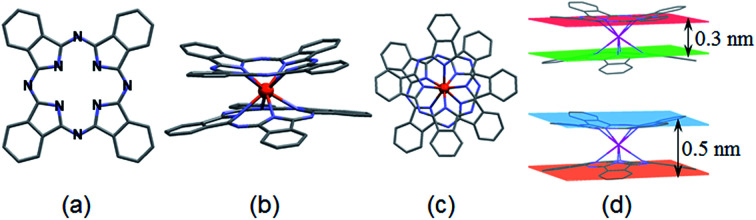

## 1. Introduction

The best approach to guaranteeing the protection of carbon steel pipelines is to hinder corrosion before it occurs, and organic coatings have long been used for that goal.^1–4^ Organic coatings protect carbon steel pipelines against corrosion by forming a physical barrier that prevents oxygen, water and corrosive ions from reaching the surface of the pipelines.^5,6^

The two major drawbacks of organic coatings are coating permeability and weak adhesion.^7^ Augmenting the adhesion of the organic coating and decreasing the resin pore channels improve the overall performance of the coatings. This extra shielding is typically achieved by incorporating nano sized pigments, which decrease the coating permeability and enhance the coating adhesion.^8–10^

Recent years have witnessed many efforts to increase the corrosion protection efficiency of organic coating by formation nano-composites formulas.^11,12^ These efforts include adding inorganic and organic particles.^13–15^ Our previous contribution in this field was the application of metal phthalocyanine pigments to improve the corrosion protection performance of the epoxy/metallophthalocyanine nanocomposite coatings.^16^ In the present study, lanthanide metal bis-phthalocyanine complexes (LnPc_2_) had been used to obtain novel nanocomposite coatings to provide excellent corrosion protection to the underlying carbon steel pipelines. The investigation of the new materials performance involved electrochemical, water permeability and pull-off adhesion SEM, TGA, scratch hardness, impact resistance, bend test and contact angle analysis.

## 2. Methods

### 2.1. Materials

Carbon steel plates (supplied from Alex. National Iron and Steel Co.) with the following composition (wt%): 0.02 Cu; 0.012 Ni; 0.7 Mn; 0.005 P; 0.06 C; 0.001 S; 0.002 V; 0.004 Mo; 0.015 Cr; 0.06 Si and balanced Fe, were employed as metallic substrates.

Alkyd resin (middle oil length resin, solid content = 50%, viscosity = 2000 mPa s, ATCOAT company), the hardener (isophoronediamine, BASF Co.) and xylene (Purechem Co.) were industrial grade materials and were used as purchased. Hydrochloric acid (35%) used in the study were purchased from Sigma-Aldrich.

### 2.2. Synthesis of LnPc_2_

**Fig. 1:**
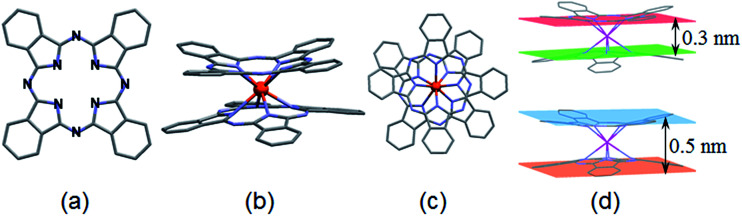
(a) The phthalocyanine macrocycle (Pc = C_32_H_16_N_8_) with N atoms highlighted; framework size 1.3 × 1.3 nm^2^. (b) LnPc_2_ double-decker molecular structure side view, and (c) top view; (d) maximum distances, between planes determined (top) by the inner pyrrole nitrogen atoms and (bottom) between planes determined by the outer benzene ring frames (estimated based on original Cambridge Crystallographic Data files).

The LnPc_2_ complexes (Ln = Pr, Sm, Ho) were all synthesized at the Faculty of Chemistry, Opole University (Poland) according to an original procedure reported elsewhere.^17^ The lanthanide metals (Ln) typically form double-decker complexes including the trivalent Ln(3+) ion sandwiched between two phthalocyanine ligands. The molecular structure of LnPc_2_ along with some average size data have been shown in Fig. 1 and the compounds identity was previously confirmed by UV-Vis and FTIR spectroscopy.^17^

### 2.3. Preparation of alkyd@LnPc_2_ nanocomposite coatings

The solvent (xylene), LnPc_2_ powder (1.0%) and dispersant agent (Polyetherphosphate, Evonik Nutrition & Care GmbH) were mixed and ground to avoid the agglomeration of nanoparticles. Such prepared mixture was incorporated into the alkyd resin (75%). The final composite was mixed using a high speed mechanical stirrer followed by ultrasonication (1 h) and then ground for 2 h to obtain the desired fineness. The hardener was added to the coating composite in the ratio of 1 : 3.

The carbon steel plates were cut into 3.0 × 1.5 cm^2^ pieces. The test plates were pre-treated by mechanical cleaning, degreasing in acetone and rinsing with distilled water before being coated. The nanocomposite coatings were applied on the surface by dip-coating on pretreated carbon steel plates. The dry film thicknesses of the coatings (35 ± 10 μm) were measured by a micrometer (B.C. Ames Co.). For the sake of comparison, a number of experiments were conducted with the neat alkyd coating only, without the addition of LnPc_2_.

### 2.4. Electrochemical experiments

Electrochemical experiments were recorded in 3 electrodes mode using an ACM potentiostat (Gill AC) controlled with the Z-View program software. A platinum mesh was used as counter electrode and the reference electrode was a saturated calomel electrode (SCE) and all potentials were referenced *versus* the SCE.

EIS measurements were conducted after 7 days of immersion, at open circuit potential (OCP) in the frequency range of 30 kHz to 0.01 Hz, with perturbation amplitude of 10 mV.

The potentiodynamic polarization experiments were carried out in the potential range of (−250 mV) to (+250 mV) *vs.* OCP with a sweep rate of 0.166 mV s^−1^.

Each experiment was repeated at least three times under practically identical conditions to provide good reproducibility for the results.

### 2.5. Characterization experiments

The morphology of the selective samples before and after the corrosion analysis was observed using a scanning electron microscopy (SEM: JEOL).

Pull-off adhesion tester (PAT model GM01/6.3 kN) was used to investigate the adhesion strength of coatings according to the ASTM D4541 standard method.

Scratch hardness for coatings was performed according to ASTM D7027 using Elcometer 3025 Scratch/Shear Tester.

Impact resistance for coatings was performed according to ASTM D2794 using BYK-Gardner ISO Impact Tester.

The bend test was performed in accordance with ASTM D522 using TQC Bend Test Conical Mandrel.

The contact angle test between water and coatings surface was measured using OCA 15EC (Dataphysics) in accordance with ASTM D7334.

The TGA analysis of the coatings was carried out using TGA 550 TA Instruments in accordance with ASTM E1131, within the temperature range 10–800 °C, at a heating rate of 10 °C min^−1^ in a nitrogen atmosphere.

## 3. Results and discussion

### 3.1. Electrochemical studies

**Fig. 2:**
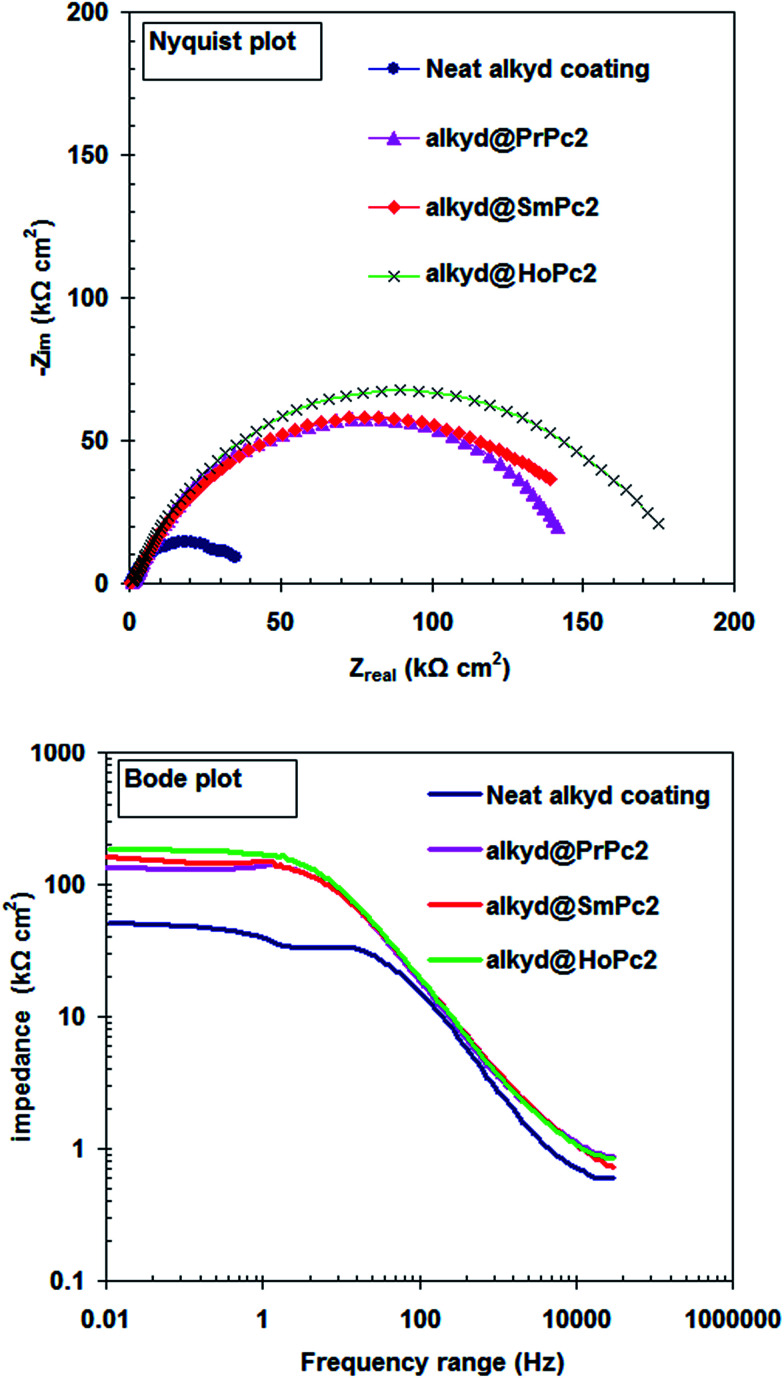
EIS plots for the carbon steel coated with the alkyd resin only and when including the LnPc_2_ after 7 days of storage in 0.5 M HCl solution at 298 K.

Fig. 2 depicts the electrochemical impedance spectroscopy approach (Nyquist and Bode module) used to study the influence of LnPc_2_ on the corrosion performance of nanocomposite coatings. The EIS plots (Fig. 2) were recoded for carbon steel coated with the alkyd resin only and when including the PrPc_2_, SmPc_2_ and HoPc_2_, after 7 days of storage in 0.5 HCl solution at 298 K.

**Fig. 3:**
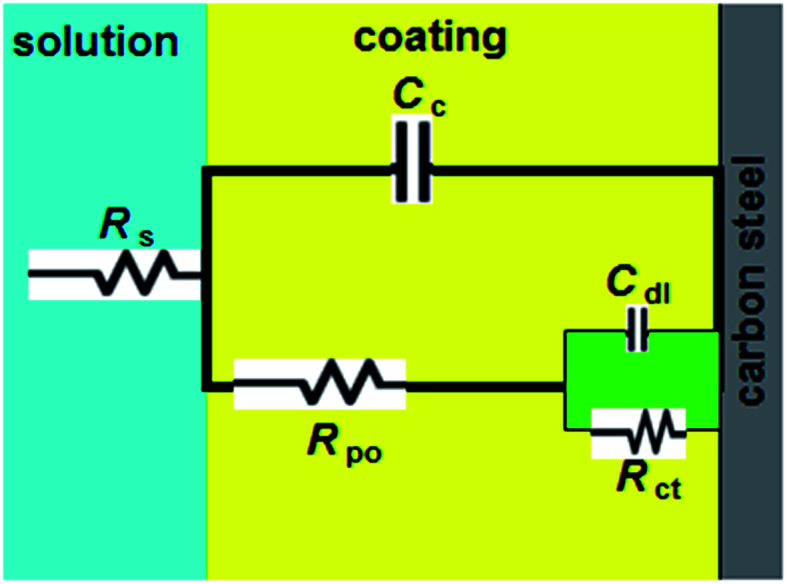
Equivalent circuit for the neat alkyd coating.

Generally, a carbon steel surface protected with an undamaged coating layer has very high impedance values. The neat alkyd coating degrades with time, resulting in more complex impedance behavior. The Nyquist plot for neat alkyd coating is characterized by two well-defined time constants (see Fig. 2). After a certain period of time, the electrolyte penetrates into the coating and forms a new liquid/metal interface under the coating.^18^ A corrosion process can occur at this new interface.^19^ The simple equivalent circuit for neat alkyd coating was schematized in Fig. 3.^20^ In this equivalent circuit, *R*_s_ is the solution resistance, *C*_c_ represents the capacitance of the intact coating, *R*_po_ (pore resistance) is the resistance of ion conducting paths that have been developed within the coating. On the carbon steel surface side, the corrosive solution penetrates through the defective neat alkyd coating. The interface between this corrosive solution and the surface of carbon steel is modeled as a double layer capacity (*C*_dl_) in parallel with charge transfer resistance (*R*_ct_). All the equivalent circuit parameters are listed in Table 1. The total impedance at low frequency is the sum of *R*_s_, *R*_po_ and *R*_ct_, while the total impedance at the point between two semi-circles is the sum of *R*_s_ and *R*_po_.^21^ The semi-circle in the high frequency region is due to the coating film and the second semi-circle at the low frequency region is due to the double layer capacitance.^22^

**Table 1:** EIS parameters for carbon steel coated by alkyd resin only and when including the LnPc_2_ particles, immersed in 0.5 M HCl solution at 298 K

Coating type	*R*_po_ kΩ cm^2^	*C*_c_ × 10^−8^ F cm^−2^	*R*_ct_ kΩ cm^2^	*C*_dl_ × 10^−8^ F cm^−2^	*η*_R_%
Neat alkyd coating	21.8	73.5	14.6	39.2	—
alkyd@PrPc_2_	140.2	5.6	—	—	84.3
alkyd@SmPc_2_	151.4	5.2	—	—	85.6
alkyd@HoPc_2_	173.2	4.6	—	—	87.4

**Fig. 4:**
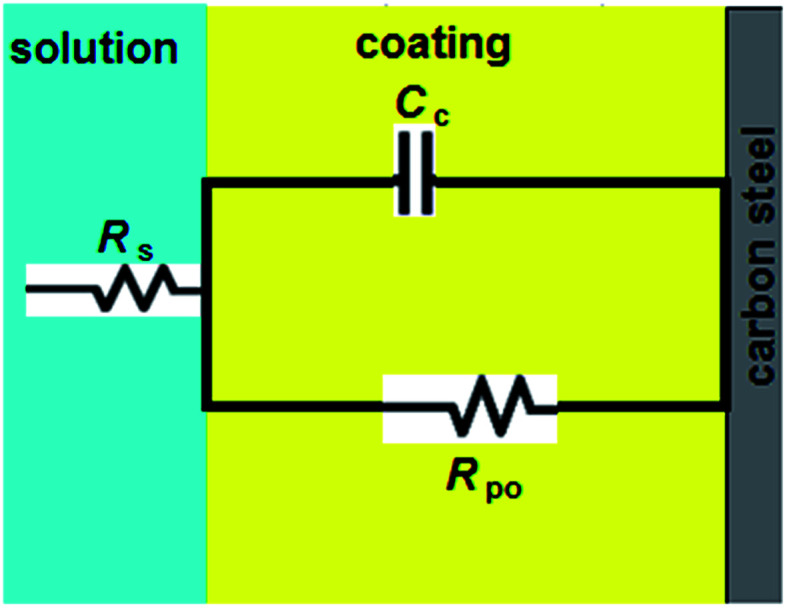
Equivalent circuit for the alkyd@LnPc_2_ coatings.

Upon incorporating of the LnPc_2_ additives with alkyd coating, a high performance coating with excellent barrier properties was formed. At this stage, *R*_po_ is extremely high. In this case, the Nyquist plot exhibits the characteristic semi-circle of a Randles-like cell which may be due to the resistance and capacitance of the coating (see Fig. 4).

When comparing the *R*_po_ and *C*_c_ values obtained from different coatings (see Table 1) it is clear that the barrier effect of a coating increases in the following order: PrPc_2_ < SmPc_2_ < HoPc_2_.

The percent protection efficiency (*η*_R_%) (see Table 1) is related to *R*_po_, by the formula:1
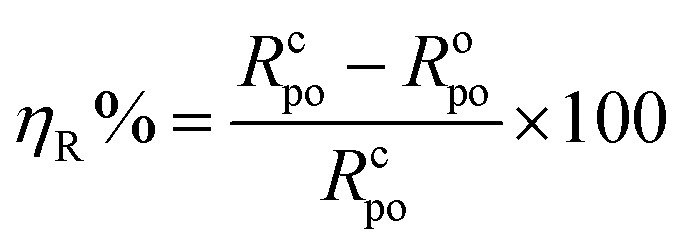
where *R*^o^_po_ and *R*^c^_po_ represent pore resistance for carbon steel coated by alkyd resin only and when including the LnPc_2_ particles, respectively.

We can see from Table 1 that the alkyd@LnPc_2_ nanocomposite coatings provided improved protection efficiency *η*_R_% in acid solution. This confirms that the alkyd@LnPc_2_ nanocomposite coatings were effective in protecting the carbon steel when exposed to a 0.5 M HCl solution.

**Fig. 5:**
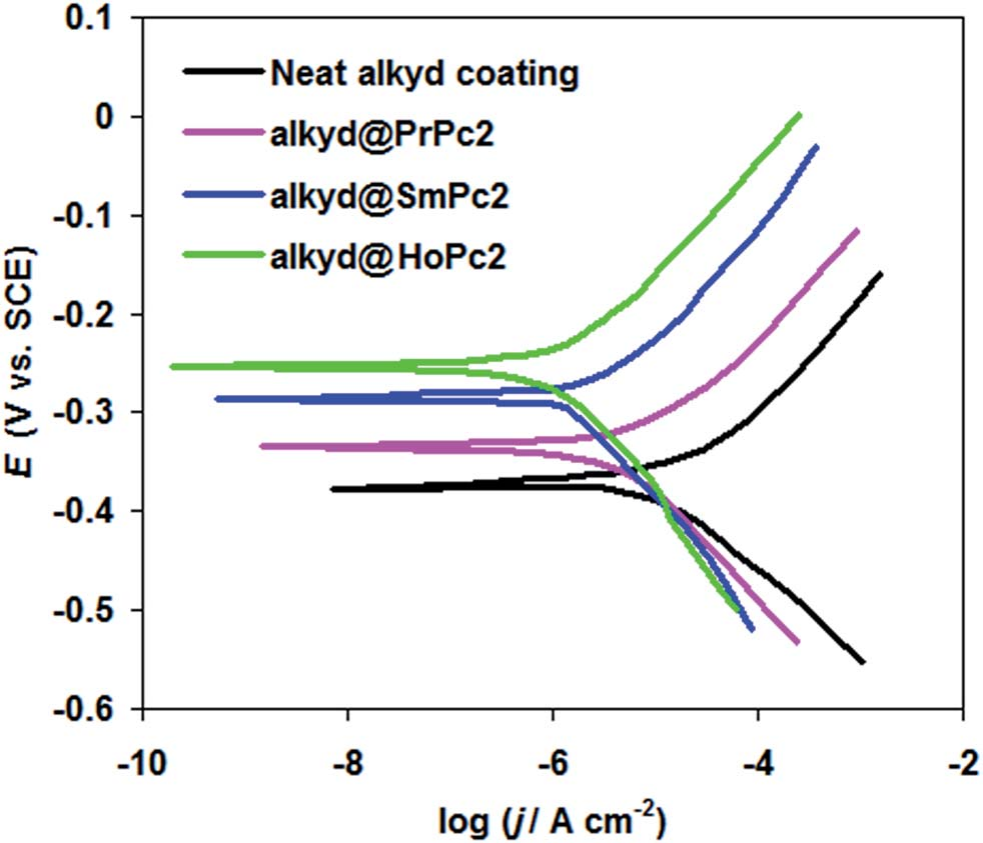
Potentiodynamic polarization curves for the carbon steel coated with the neat alkyd and alkyd@LnPc_2_ nanocomposite coatings in 0.5 M HCl solution at 298 K.

To corroborate EIS data, the effect of alkyd@LnPc_2_ nanocomposite coatings on carbon steel corrosion behavior was investigated in a 0.5 M HCl solution using potentiodynamic polarization (see Fig. 5).

Major polarization parameters, corrosion current density (*j*_corr_), corrosion potential (*E*_corr_), and inhibition efficiency (*η*_j_%) are presented in Table 2.

The inhibition efficiency (*η*_j_%) is related to corrosion current density (*j*_corr_), by the formula:2
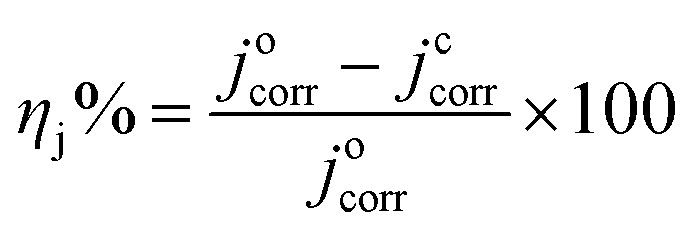
where *j*^o^_corr_ and *j*^c^_corr_ represent the corrosion current density for carbon steel coated by the alkyd resin only and when including the LnPc_2_ particles, respectively.

From Table 2 it is clear that carbon steel coated by the alkyd@LnPc_2_ nanocomposite coatings exhibits a decreased *j*_corr_, as well as an increased *E*_corr_, compared with neat alkyd coating. Further, it was found that the alkyd resin containing LnPc_2_ particles showed better inhibition efficiency *η*_j_% than the neat alkyd coating (see Table 2).

**Table 2:** The values of corrosion current density, corrosion potential and the corresponding inhibition efficiency for carbon steel coated by alkyd resin only and when including the LnPc_2_ particles, immersed in 0.5 M HCl solution at 298 K

Coating type	*−E*_corr_ V *vs.* SCE	*j*_corr_ A cm^−2^	*η*_j_%
Neat alkyd coating	0.377	18.97 × 10^−6^	—
alkyd@PrPc_2_	0.333	2.40 × 10^−6^	87.3
alkyd@SmPc_2_	0.285	1.76 × 10^−6^	90.7
alkyd@HoPc_2_	0.253	1.40 × 10^−6^	92.6

### 3.2. Absorption of H_2_O studies

H_2_O has a higher dielectric constant (*ε*_H_2_O_ = 80) than that of the alkyd one, so the value of *C*_c_ with absorbed H_2_O is higher than that of the dry coating.^23^ Information available on the amount of water absorbed by the coating at different stages of exposure (*∅*) is provided by the Brasher–Kingsbury equation (see eqn (3)).^24^

3 *∅* = log(*C*_*t*_/*C*_0_)/log *ε*_H_2_O_

here *C*_*t*_ is the coatings capacitance at time *t*, *C*_0_ is the initial coatings capacitance, and *ε*_H_2_O_ is the dielectric constant of H_2_O (*ε*_H_2_O_ = 80).

**Fig. 6:**
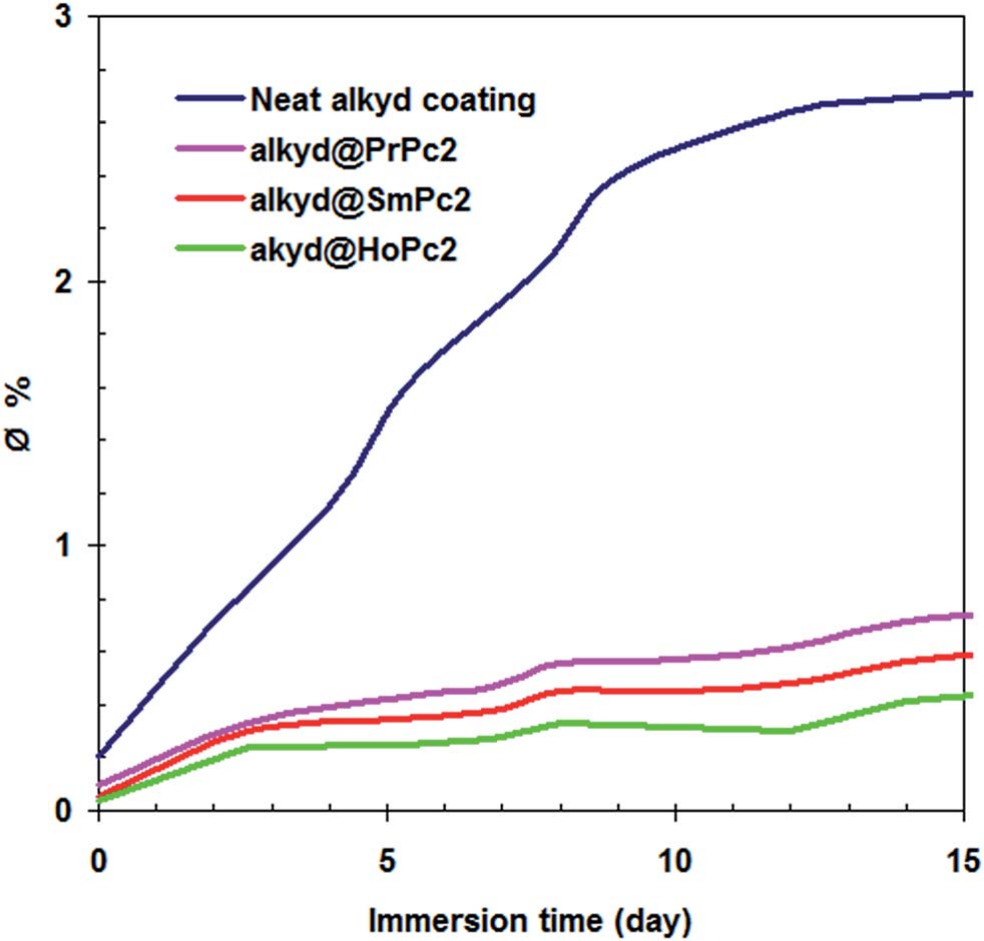
The water absorption (*∅*) featured by the investigated coatings as a function of immersion time.

The coating capacitance can be measured at different immersion time by fitting the equivalent circuit to the EIS data. Fig. 6 reports on the amount of water (*∅*) absorbed by the various coatings during different immersion times. It was observed that the alkyd@LnPc_2_ nanocomposite coatings gave a lower *∅* value, when compared to the neat alkyd coating. This would have indicated that the lanthanide bis-phthalocyanine compounds prevent the passage of water and corrosive ions throughout the coating layers. The permeability compared to the neat alkyd coating is considerably reduced by the use of LnPc_2_. A possible explanation one may relate to blocking of the resin pores by the LnPc_2_ particles and/or repelling of water molecules by the highly hydrophobic LnPc_2_ species. The lowest water absorption was demonstrated by the alkyd/HoPc_2_ nanocomposite coating.

### 3.3. Effect of LnPc_2_ on physico-mechanical properties

The adhesion strength of coating is known to play a major role in enhancing of the corrosion protection performance of coatings.^25^

**Fig. 7:**
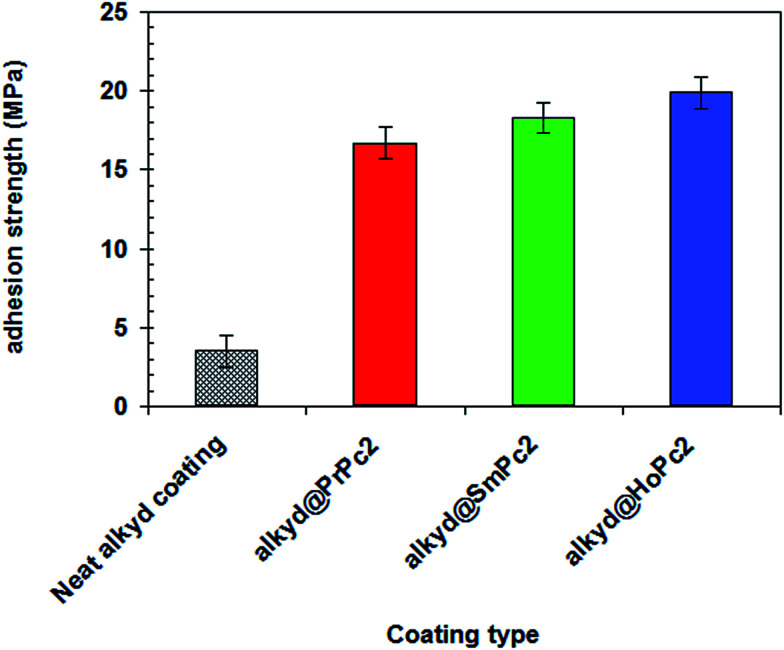
The effect of LnPc_2_ on the pull-off adhesion strength of alkyd resin-based coatings.

The effect of LnPc_2_ on the adhesion strength of alkyd coating was evaluated by pull-off adhesion strength experiments and the results were shown in Fig. 7. As follows from there, incorporation of LnPc_2_ into the alkyd coating effectively enhanced the adhesion strength of coating. The average adhesion strength of the neat alkyd coating (without LnPc_2_) was 3.34 MPa, while significantly higher values of 16.73 MPa, 18.34 MPa and 19.94 MPa were obtained for alkyd@PrPc_2_, alkyd@SmPc_2_ and alkyd@HoPc_2_ composites, respectively. Collectively, these results establish the ability of LnPc_2_ particles to increase the resistance of a coating to separation from a substrate when a perpendicular tensile force is applied.

The effects of the LnPc_2_ particles on scratch hardness, impact resistance, bend test and contact angle for alkyd coating behavior were examined and the results are listed in Table 3.

**Table 3:** Physico-mechanical properties for neat alkyd and alkyd@LnPc_2_ nanocomposite coatings

Coating type	Scratch hardness (kg)	Impact resistance (kg cm^2^)	Bend test	Contact angle
Neat alkyd coating	3.5	75	Pass	92°
alkyd@PrPc_2_	9.0	110	Pass	98°
alkyd@SmPc_2_	10.5	131	Pass	99°
alkyd@HoPc_2_	12.2	115	Pass	101°

Interestingly, the scratch hardness of the coatings increased by incorporation of LnPc_2_ particles into the coating. The best scratch hardness was gained by the alkyd@HoPc_2_ composite. This improvement in scratch hardness may possibly be related to the restriction of indentation due to the increase in physical interactions between the alkyd resin and LnPc_2_ particles.^26^

Furthermore, the impact resistance of the alkyd resin coating became evidently improved by doping it with LnPc_2_ (Table 3). Presumably incorporation of LnPc_2_ particles within the alkyd resin matrix restricts the chain mobility, thus leading to high impact resistance.^26^

Excellent bend test results were observed also for coatings without film cracks.

The hydrophobic nature of the alkyd@LnPc_2_ nanocomposites was further characterized through contact-angle measurements. Contact angles determined for the neat alkyd coating, alkyd@PrPc_2_, alkyd@SmPc_2_ and alkyd@HoPc_2_ were 92°, 98°, 99° and 101°, respectively, indicating that all of the alkyd@LnPc_2_ coatings were hydrophobic by nature. This means that LnPc_2_ particles decrease the contact of the coating surface with the corrosive solution leading to an improvement in the anticorrosion behavior of the coatings.^27^

**Fig. 8:**
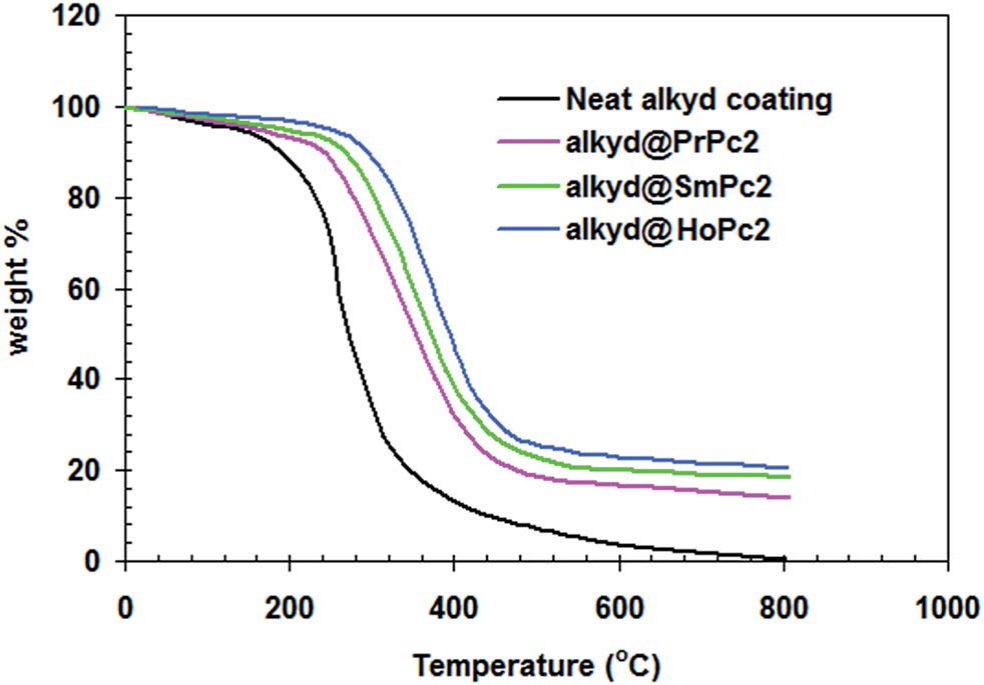
TGA plot of alkyd@LnPc_2_ nanocomposite coatings.

Fig. 8 displays the results of the thermogravimetry analysis (TGA) of the studied coatings. Pure alkyd coating was completely decomposed (100%) at 800 °C, while the alkyd@LnPc_2_ samples were less decayed (80 ± 5%). This would suggest the addition of LnPc_2_ into the resin matrix improved the thermal stability of the coatings, presumably due to interaction between LnPc_2_ particles and the alkyd resin.^28^

### 3.4. Morphology studies

**Fig. 9:**
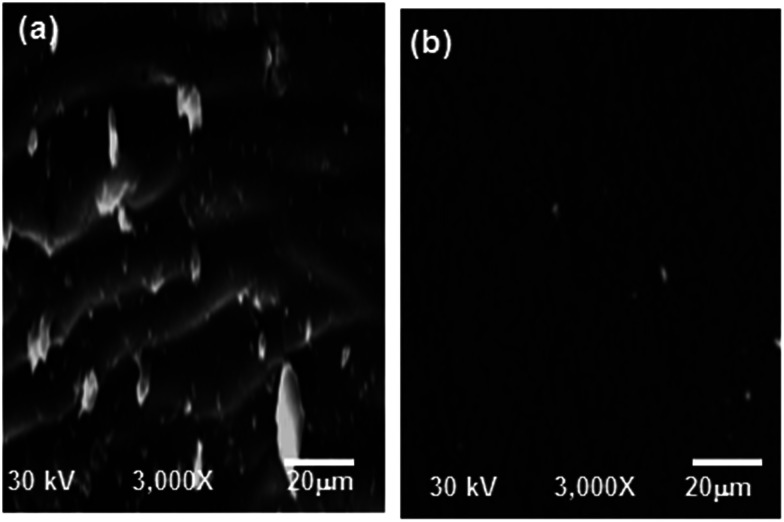
SEM micrographs of coated carbon steel immersed in 0.5 M HCl solution at 298 K for (a) neat alkyd coating and (b) alkyd@LnPc_2_ nanocomposite coatings.

Scanning electron microscopy (SEM) was used to investigate the morphology of the surface coated by the alkyd@LnPc_2_ envelope. The two carbon steel samples coated by the alkyd resin and the alkyd@LnPc_2_ nanocomposites were exposed to a 0.5 M HCl solution for 168 h. The SEM images are presented in Fig. 9. In the case of the carbon steel coated only by the resin (Fig. 9a), a patterns of small cracks were observed over the coating layer. It is clear then why the coating layer was severely damaged by the corrosive solution. In contrast, the alkyd@LnPc_2_ coatings (Fig. 9b), featured a much better surface morphology, indicating for a considerable improvement of the coating layer texture by adding LnPc_2_.

### 3.5. Explanation of the role of LnPc_2_

Alkyd@LnPc_2_ nanocomposite coatings are a class of materials in which LnPc_2_ particles with nanoscale dimensions are embedded in an alkyd resin matrix.^29^ The small size of LnPc_2_ particles allowing their even dispersion within the resin matrix may be considered an important issue determining the unique properties of composites involving such compounds.^30^

The general idea concerned with the use of LnPc_2_ components to produce anti-corrosion coatings was to create a synergy between the various constituents, so that novel properties capable of meeting or exceeding design expectations could be achieved.

In general, alkyd@LnPc_2_ nanocomposite coatings feature diverse mechanical and electrochemical properties which are different from those of the neat alkyd coating.

The corrosion process in the acid solutions that occurs on the carbon steel surface involves oxidation reaction (Fe_(s)_ → Fe_(aq)_^2+^ + 2e) and one or more reduction reactions.^31–34^ These reactions occur at the metal surface where a corrosion cell has been established. To reduce the corrosion rate it is necessary to control the dynamics of the corrosion process by applying neat alkyd coating on the metal. Such a coating acts as a barrier between the carbon steel and its environment, slowing down the rate at which water, oxygen, or ions from the environment reach the metal surface.^35^ In the alkyd@LnPc_2_ nanocomposite coatings, the nano-size particles of LnPc_2_ are well dispersed in the alkyd resin, thus being able to prevent the diffusion of water, oxygen, and ionic species through the spaces occurring in the resin's matrix.

LnPc_2_ particles may effectively block the resin's pores (at least to some extent) and in this way reduce the permeability of the nanocomposite. Besides, LnPc_2_ phthalocyanines are highly hydrophobic and therefore their molecules have a natural potential to repel water species as well as ions solvated by H_2_O. Such effects should have reasonably improved the coating's protection efficiency.

The principal reason, which may elucidate the strong adhesion of alkyd@LnPc_2_ nanocomposite coatings is definitely related to the magnetic properties of Ln(3+) ions and hence obviously featured by the LnPc_2_ particles. Open f-shell cations, including unpaired electrons, are strongly paramagnetic under normal conditions (and some of them become even ferromagnetic,^36^) and it is very possible they could effectively interact with the ferromagnetic atoms of iron (of the carbon steel) *via* magnetic field forces. From among the rare earth metals, Ho has the highest magnetic permeability, and the Ho^3+^ ion exhibits the largest magnetic moment (*μ*_eff_, B.M.), Table 4. Incidentally, the ferromagnetic performance revealed by the holmium metal is even far better than that of Fe.^36^ Hence, this particular paramagnetic feature is certainly dominant among the factors that might have contributed to reinforcing the contact between the alkyd matrix and the steel core. This conclusion was plausibly confirmed in the pull-off tests which showed the adhesion strength of the alkyd@HoPc_2_ nanocomposite was 6 times as large as found for the alkyd resin alone (Fig. 7).

**Table 4:** Effective magnetic moments, *μ*_eff_ (B.M.) of the Ln^3+^ ions involved in the investigated LnPc_2_ compounds (experimental data, according to (ref. 33))

Ln^3+^	Pr^3+^	Sm^3+^	Ho^3+^
*μ*_eff_ (B.M.)	3.6	1.7	10.7

It must be emphasized, that the statement postulated above well correlates with the results reported in Fig. 2, 5 and 6. From the Nyquist and Bode plots (Fig. 2) follows, that the corrosion mechanism in the respective alkyd@LnPc_2_ coatings seems quite similar, despite some differences in *R*_po_ values (averaged increment of 9%). On the other hand, in the case of the water absorption process, the differences in *∅* are more pronounced, but follow the same sequence of coating quality (Fig. 6). Presumably, both *R*_po_ and *∅* values must somehow be related to the adhesion strength of the coating, which determines its stability and hence also the principal properties. Undoubtedly, the paramagnetism of the lanthanides is crucial here, although it is difficult to give a clear-cut explanation for the differences observed between the studied systems. Most probably these may arise from more specific electronic effects occurring at the atomic quantum level, such as the strong spin–orbit coupling featured by the rare earth elements. One may assume, that interaction with the magnetic field generated by the ferromagnetic domains of iron may impose diverse quantum resonance within the “coating–carbon steel” system. Hence, the sequence of the protection efficiency, as evinced by the Pr and Sm composites, must not necessarily comply with the trend demonstrated by the individual magnetic moments, as reported in Table 4.

## 4. Conclusions

Anti-corrosion properties of alkyd resin-based coatings can be improved considerably by the addition of LnPc_2_ compounds. The newly developed alkyd@LnPc_2_ nanocomposite coatings provided excellent protection for carbon steel pipelines. When compared to the neat alkyd matrix, incorporation of LnPc_2_ particles into the coating system decreases its water permeability and enhances the corrosion resistance and physico-mechanical properties. Moreover, the applied LnPc_2_ dopants significantly raised the adhesion strength of the coating. The experimentally determined protection efficiency evidently results from the magnetic properties of the particular Ln^3+^ ions, and increases in the following order: PrPc_2_ < SmPc_2_ < HoPc_2_. This finding may be considered an important progress in the search for sophisticated anti-corrosion composite systems.

## Conflicts of interest

There are no conflicts to declare.

## Supplementary Material
